# Lung volume assessment for mean dark-field coefficient calculation using different determination methods

**DOI:** 10.1186/s41747-025-00593-y

**Published:** 2025-05-23

**Authors:** Florian T. Gassert, Jule Heuchert, Rafael Schick, Henriette Bast, Theresa Urban, Tina Dorosti, Gregor S. Zimmermann, Sebastian Ziegelmayer, Alexander W. Marka, Markus Graf, Marcus R. Makowski, Daniela Pfeiffer, Franz Pfeiffer

**Affiliations:** 1 Institute for Diagnostic and Interventional Radiology, School of Medicine and Health, TUM Klinikum, Technical University of Munich (TUM), Munich, Germany; 2 Chair of Biomedical Physics, Department of Physics, School of Natural Sciences, Technical University of Munich (TUM), Munich, Germany; 3 Munich Institute of Biomedical Engineering, Technical University of Munich (TUM), Munich, Germany; 4 Division of Respiratory Medicine, Department of Internal Medicine I, School of Medicine and Health, TUM Klinikum, Technical University of Munich (TUM), Munich, Germany; 5 Munich Institute for Advanced Study, Technical University of Munich (TUM), Munich, Germany

**Keywords:** Lung volume measurement, Radiography (thoracic), Respiratory function tests, Thorax, Tomography (x-ray computed)

## Abstract

**Background:**

Accurate lung volume determination is crucial for reliable dark-field imaging. We compared different approaches for the determination of lung volume in mean dark-field coefficient calculation.

**Methods:**

In this retrospective analysis of data prospectively acquired between October 2018 and October 2020, patients at least 18 years of age who underwent chest computed tomography (CT) were screened for study participation. Inclusion criteria were the ability to consent and to stand upright without help. Exclusion criteria were pregnancy, lung cancer, pleural effusion, atelectasis, air space disease, ground-glass opacities, and pneumothorax. Lung volume was calculated using four methods: conventional radiography (CR) using shape information; a convolutional neural network (CNN) trained for CR; CT-based volume estimation; and results from pulmonary function testing (PFT). Results were compared using a Student *t*-test and Spearman *ρ* correlation statistics.

**Results:**

We studied 81 participants (51 men, 30 women), aged 64 ± 12 years (mean ± standard deviation). All lung volumes derived from the various methods were different from each other: CR, 7.27 ± 1.64 L; CNN, 4.91 ± 1.05 L; CT, 5.25 ± 1.36 L; PFT, 6.54 L ± 1.52 L; *p* < 0.001 for all comparisons. A high positive correlation was found for all combinations (*p* < 0.001 for all), the highest one being between CT and CR (*ρ* = 0.88) and the lowest one between PFT and CNN (*ρ* = 0.78).

**Conclusion:**

Lung volume and therefore mean dark-field coefficient calculation is highly dependent on the method used, taking into consideration different positioning and inhalation depths.

**Relevance statement:**

This study underscores the impact of the method used for lung volume determination. In the context of mean dark-field coefficient calculation, CR-based methods are more desirable because both dark-field images and conventional images are acquired at the same breathing state, and therefore, biases due to differences in inhalation depth are eliminated.

**Key Points:**

- Lung volume measurements vary significantly between different determination methods.
- Mean dark-field coefficient calculations require the same method to ensure comparability.
- Radiography-based methods simplify workflows and minimize biases, making them most suitable.

**Graphical Abstract:**

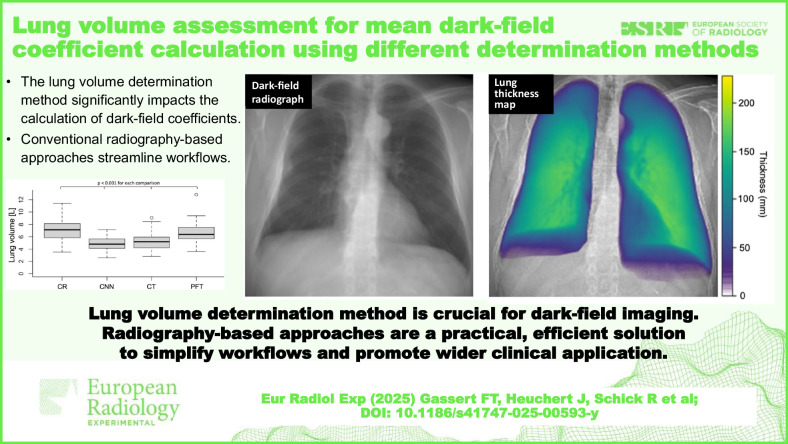

## Background

Dark-field radiography is an emerging technique that exploits the wave properties of x-rays [1]. As small-angle scattering, which occurs at interfaces between different materials, generates a dark-field signal, the healthy lung with its alveolar structure and numerous tissue-air interfaces has a relatively high signal, while surrounding homogeneous tissues generate hardly any signal [2, 3]. It has been shown that the dark-field signal reduces when the alveolar structure is impaired, as a lower number of tissue-air interfaces results in less small-angle scattering. This accounts for both diseases in which the number of alveolar walls is reduced (including pulmonary emphysema [4–6], combined pulmonary fibrosis and emphysema [7], and lymphangioleiomyomatosis [8]) as well as diseases in which the alveolar space is filled with inflammatory fluid (including COVID-19 [9] and other pneumonias [10, 11]).

Dark-field radiography has been introduced in humans only recently, with the so-called mean dark-field coefficient being an associated metric [3, 4, 9]. It is calculated as the ratio between the total dark-field signal (TDF) and the lung volume of the subject. Thus, it normalizes dark-field signal by lung size, which is essential for a fair comparison of quantitative dark-field images between different subjects, as subjects with bigger lungs tend to have a higher TDF. In a study of 40 healthy subjects, it has been shown that the mean dark-field coefficient is independent of gender, age, and height [3].

To our knowledge, all dark-field studies in humans until today have used the approach suggested by Pierce et al for the determination of the lung volume in mean dark-field coefficient calculation [12]. In this method, the lung volume is calculated using shapes derived from posteroanterior and lateral radiographs and simple equations. In principle, lung volume equals the volume of the chest minus the volumes of the heart, spine, and subphrenum. The big advantage of this method is that the necessary conventional radiography (CR) is automatically obtained with every dark-field radiography. Therefore, no additional image acquisition is needed for the mean dark-field coefficient calculation. Also, inspiration levels for dark-field radiographs and CRs are exactly the same. However, this method has only been evaluated in 35 subjects and has only been compared to whole-body plethysmography [12].

Therefore, the purpose of our study was to analyze and compare various approaches for the determination of lung volume to be used for mean dark-field coefficient calculation.

## Methods

### Participants

**Fig. 1:**
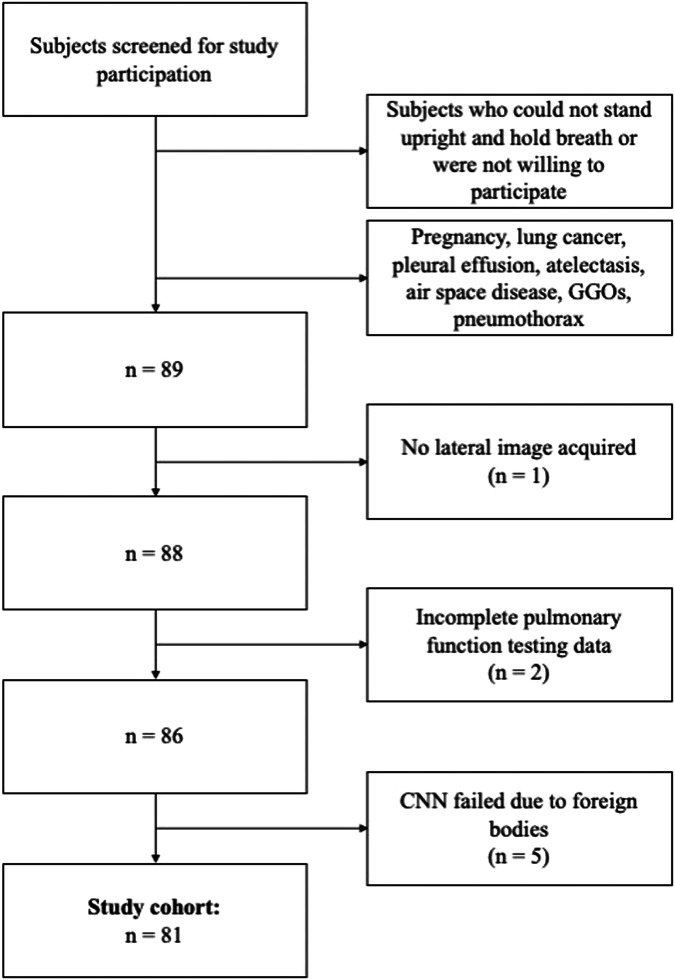
Flowchart shows participant selection. Between October 2018 and October 2020, a total of 81 participants were included in the study. *GGOs* Ground-glass opacities

This retrospective analysis of prospectively acquired data was conducted in accordance with the Declaration of Helsinki (as revised in 2013). Approval of the Institutional Review Board and the national radiation protection agency was obtained prior to this study (Ethics Commission of the Medical Faculty, Technical University of Munich, Germany; Reference Number 166/20S). Participants gave their written informed consent. Between October 2018 and October 2020, patients of at least 18 years of age who underwent chest CT as part of their diagnostic workup were screened for study participation. Inclusion criteria were the ability to consent and the ability to stand upright without help. Exclusion criteria were pregnancy, lung cancer, pleural effusion, atelectasis, air space disease, ground-glass opacities, and pneumothorax. Participants with missing lateral image, incomplete pulmonary function testing (PFT) data, and failed convolutional neural network (CNN) (see below) due to foreign bodies were also excluded. Figure 1 shows the study flow and selection process. Forty participants were previously described in a study by Gassert et al [3] evaluating the characteristics of dark-field chest x-ray imaging in healthy humans. Seventy-two participants were also studied by Willer et al [5] and Urban et al [4], assessing the diagnostic accuracy of dark-field imaging for emphysema diagnosis.

### Dark-field imaging

Dark-field imaging was performed as described previously [3–5]. In brief, dark-field images were acquired with a prototype system that uses a conventional imaging system with a diagnostic x-ray tube (MRC 200 0508 ROT-GS 1003; Philips Medical Systems, Hamburg, Germany) operated at 70 kVp and a flat-panel detector (PIXIUM 4343 F4). A three-grating interferometer inserted in the beam path enables the simultaneous acquisition of conventional attenuation radiographs and complementary dark-field images [1].

All participants were examined with one acquisition each in posteroanterior and lateral orientation at full inspiration. The acquisition time was about 7 s. The effective median dose was 37 μSv for the posteroanterior images [13] and 46 μSv for the lateral images. The entire lung was segmented in posteroanterior images and the TDF was calculated.

The dark-field coefficient has been established as the ratio between the TDF and the lung volume of an individual subject:

$${dark\; field\; coefficient}=\frac{{total\; dark\; field\; signal}}{{lung\; volume}}$$

### CT imaging

CT imaging was performed on one of three scanners: iCT or IQon Spectral, Philips Medical Systems, Hamburg, Germany; SOMATOM go.Top, Siemens Healthineers) with routine clinical protocols. Images were reformatted with 3-mm slice thickness using a lung-specific convolution kernel.

### Lung volume determination methods

For this study, the lung volume was derived using four different approaches: from posteroanterior and lateral attenuation images as described by Pierce et al [12], from posteroanterior images only using a CNN, from CT images, and from PFT.

#### Lung volume derived from CRs

Using the approach adapted from Pierce et al [12], the lung volume was calculated from the posteroanterior and lateral attenuation images acquired with the dark-field chest radiography system. In brief, the lung and surrounding structures were segmented manually on images in both orientations, and the volumes were calculated using an approximation based on the formula: lung volume = chest volume minus (heart + spine + subphrenum) [12].

#### Lung volume derived from CNN

**Fig. 2:**
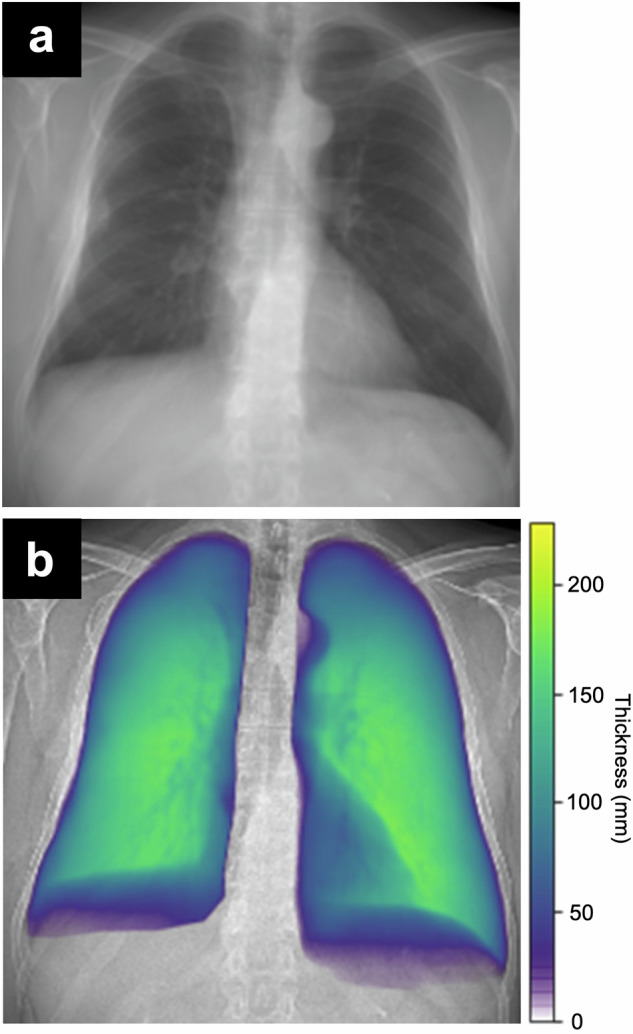
Example attenuation-based radiograph acquired with the dark-field scanner (**a**) and computed lung thickness map (**b**)

The lung volume determination by CNN was based on a model introduced by Schultheiss et al [14], which uses CT-based synthetic radiographs for training. Briefly, for the training, a U-Net architecture was used to estimate local lung thickness on 5,250 synthetic radiographs derived from CT scans, while the CT-based lung volume was used as ground truth. The model was evaluated on a test set of 131 synthetic and 45 clinical CRs [14]. The model was adapted to the dark-field scanner’s imaging and processing parameters, namely the geometry as source-to-sample/sample-to-detector distance, the simulated spectrum with 70 kVp, the detector size, thickness, and density, as well as the aluminum filter thickness. It was then applied to this study’s participants’ attenuation radiographs acquired with the dark-field scanner. Lung masks, which were selected manually for each patient on the CR from the dark-field scanner, were then applied to the local thickness prediction maps (Fig. 2) to make sure that lung volume was only calculated from actual lung tissue. In a last step, the total CNN-based lung volume was calculated by integrating the predicted lung thickness over the whole lung area.

#### Lung volume derived from CT images

Lung volume was derived from CT images using dedicated commercial software (IntelliSpace Portal, version 11.1.1; Philips), automatically segmenting the lung. Automatic segmentations excluded large airways while smaller airways were included. All segmentations were doublechecked and corrected manually if needed.

#### Lung volume derived from PFT

All participants performed a standardized PFT according to the European Respiratory Society [15, 16], providing the total lung capacity (TLC). The PFT consisted of a combination of spirometry with whole-body plethysmography (MasterScreen Body, Jaeger, Wuerzburg, Germany). TLC was considered as the surrogate of lung volume derived from PFT.

### Statistical analysis

Statistical analysis was performed using the statistical package R version 3.2.4 (R Foundation for Statistical Computing). A *p*-value < 0.05 was considered statistically significant for all tests. A Shapiro–Wilk test was performed to assess the normality of lung volume data. Since three out of four datasets showed normal distribution, we applied the Student *t*-test for group comparisons. To account for deviations from normality in the PFT data and to increase robustness against outliers, we used the nonparametric Spearman ρ for correlation analyses. This combined approach allows for both valid mean comparisons and robust correlation estimates across datasets with partially differing distribution characteristics.

## Results

### Participants

We studied 81 participants (51 men, 30 women), aged 64 ± 12 years (mean ± standard deviation), with height being 170 ± 7 cm and weight 75 ± 15 kg (Table 1).

**Table 1:** Patient demographics and lung volumes determined with four different methods

Parameters	All	Men	Women	*p*-value
Number of patients	81	51	30	−
Age	64 ± 12	66 ± 12	62 ± 11	0.21
Weight (kg)	75 ± 15	78 ± 15	70 ± 13	0.01
Height (cm)	170 ± 7	173 ± 4	164 ± 6	< 0.001
Lung volume (L) from				
Conventional radiography	7.27 ± 1.64	8.08 ± 1.42	5.90 ± 0.91	< 0.001
Convolutional neural network	4.91 ± 1.05	5.37 ± 0.93	4.14 ± 0.78	< 0.001
Computed tomography	5.25 ± 1.36	5.74 ± 1.36	4.42 ± 0.85	< 0.001
Pulmonary function testing	6.54 ± 1.52	7.23 ± 1.34	5.38 ± 1.01	< 0.001

### Comparison of different lung volume determination methods

**Fig. 3:**
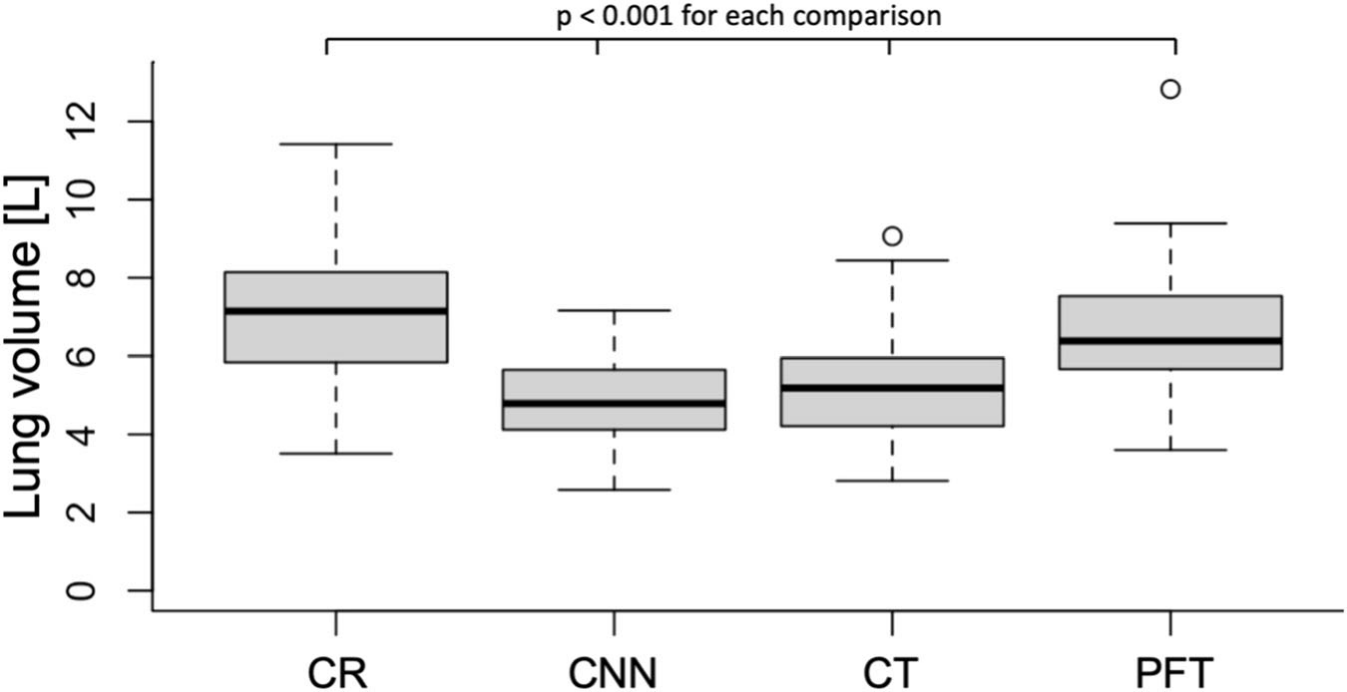
Lung volumes determined with conventional radiography (CR), convolutional neural network (CNN), computed tomography (CT), and pulmonary function testing (PFT). All lung volumes were significantly different from each other

The mean lung volume was 7.27 ± 1.64 L (mean ± standard deviation) for the CR method, 4.91 ± 1.05 L for the CNN method, 5.25 ± 1.36 L for CT imaging, and 6.54 ± 1.52 L for PFT. All lung volumes derived from the various methods were different from each other (*p* < 0.001 for all; Table 1 and Fig. 3).

**Fig. 4:**
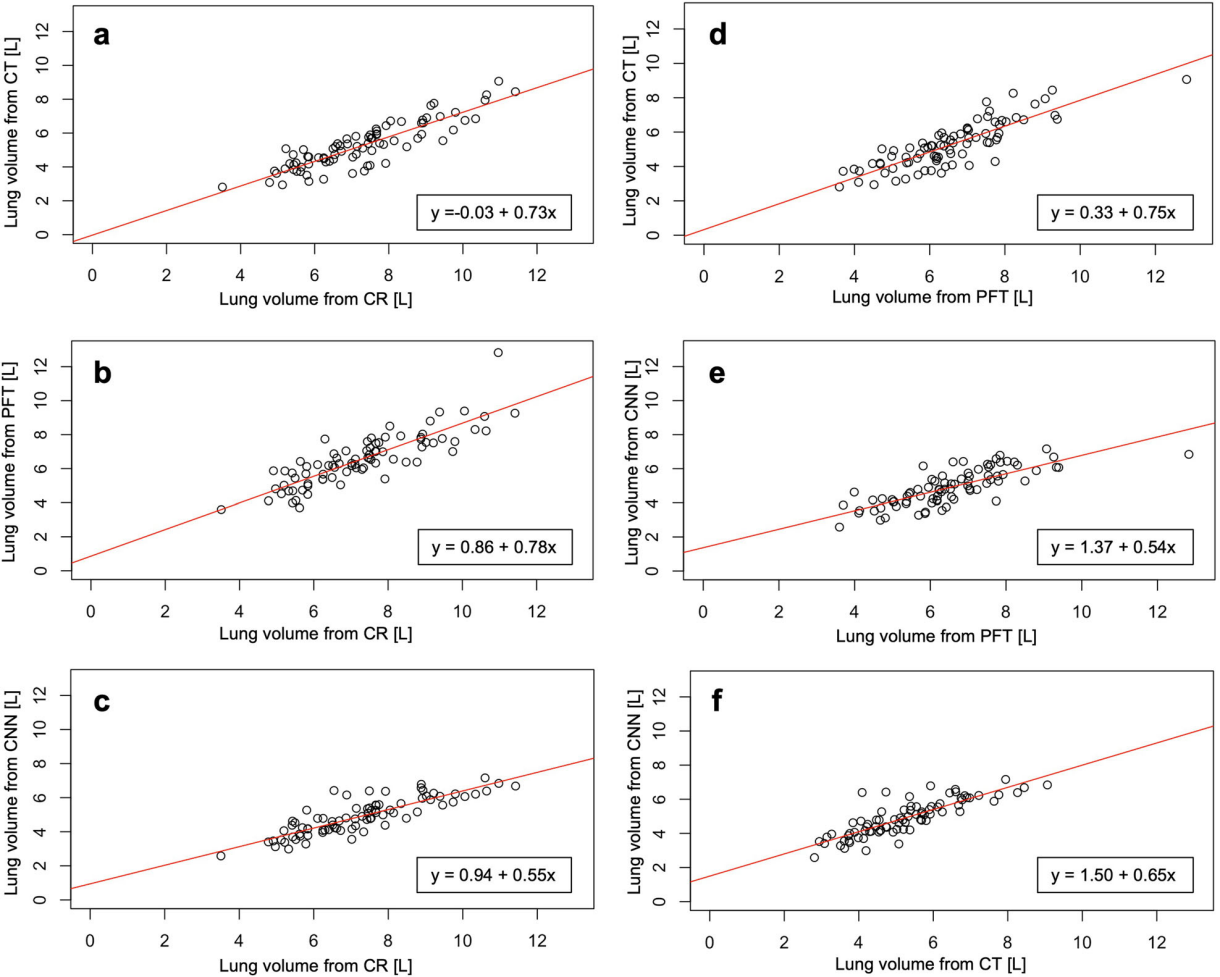
Scatter plots for the comparison of lung volumes determined with different methods: computed tomography (CT) *versus* conventional radiography (CR) (**a**); pulmonary function testing (PFT) *versus* CR (**b**); convolutional neural network (CNN) *versus* CR (**c**); CT *versus* PFT (**d**); CNN *versus* PFT (**e**); and CNN *versus* CT (**f**). Red lines indicate regression lines, and the box on the bottom right provides respective intercepts and slopes

When comparing all four lung volume determination methods with each other, a high to very high positive correlation was found for all combinations (*p* < 0.001 for all; Table 2, Fig. 4), the highest one observed between CT and CR (*ρ* = 0.88) and the lowest one between PFT and CNN (*ρ* = 0.78).

**Table 2:** Correlation between lung volumes determined from conventional radiography (CR), convolutional neural network (CNN), computed tomography (CT), and pulmonary function testing (PFT)

	CR		CNN		CT		PFT	
	Spearman ρ	*p*-value	Spearman ρ	*p*-value	Spearman ρ	*p*-value	Spearman ρ	*p*-value
CR	−	−	0.85	< 0.001	0.88	< 0.001	0.84	< 0.001
CNN	0.85	< 0.001	−	−	0.84	< 0.001	0.78	< 0.001
CT	0.88	< 0.001	0.84	< 0.001	−	−	0.84	< 0.001
PFT	0.84	< 0.001	0.78	< 0.001	0.84	< 0.001	−	−

## Discussion

In this study, we compared in the same subjects four different methods for the assessment of lung volume: a method suggested by Pierce et al [12] for computation of lung volume from conventional radiographs; a CNN trained on synthetic radiographs and applied to CR; a CT-based lung volume estimation; and PFT. The lung volume was significantly different across all four methods, but lung volumes from all methods showed a high to very high correlation with each other (*ρ* = 0.78–0.88).

While all four methods have advantages and disadvantages, CT imaging is considered the reference standard for the determination of lung volume, and PFT for the determination of the TLC. The greater mean lung volume from PFT (TLC) compared to that obtained by CT imaging in our study might be due to two reasons: first, in PFT, patients are instructed explicitly, repeatedly and with emphasis to breathe in and out by a nurse, while in CT there is only a single automatic announcement. That might lead to deeper inspiration in PFT compared to CT and thus higher values of measured lung volumes. Second, PFT is performed while sitting, and therefore, the upper body is upright, while CT imaging is performed in the supine position. This finding is in line with two studies by Yamada et al [17, 18], which have shown that lung volume from CT imaging in the standing position is greater compared to when performed in the supine position. Also, a study by Bakker et al [19] found that the CT-derived TLC was 590 ± 430 mL (mean ± standard deviation) lower compared to body plethysmography in a cohort of 100 participants. This is well within the range of our resulting lung volumes (5.25 L ± 1.36 for CT *versus* 6.54 L ± 1.52 for PFT). Bakker et al [19] also found that the lung volume difference between assessment by CT and PFT could be reduced to only 280 ± 340 mL when CT scans were performed under spirometer guidance, which, again strengthens the presumption, that the difference in inhalation depth might bias the comparison between CT and PFT, as well as the CR-based approaches following the method by Pierce et al [12] or using a CNN.

A notable finding in our study was that the CNN-derived lung volume was closer to the CT-derived lung volume estimation than to the CR-based estimation, despite both methods being applied to the same radiographs. This is reflected in our results (Table 1), where CR-derived lung volumes were much greater than those from CT (7.27 ± 1.64 L *versus* 5.25 ± 1.36 L), whereas CNN-derived lung volumes aligned more closely with CT-derived lung volumes (4.91 ± 1.05 L). This suggests that CNN-based lung volume determination may provide a more accurate structural assessment compared to the traditional CR method. However, CNN-based segmentation is still subject to biases related to training data and image quality, necessitating further validation in larger and more diverse cohorts.

Interestingly, the observed sex-based differences in lung volume were less pronounced for CNN-derived values compared to CR-based values. As seen in Table 1, the CR approach overestimated lung volume more prominently in men (8.08 ± 1.42 L for men *versus* 5.90 ± 0.91 L for women), whereas the CNN method showed a less pronounced difference (5.37 ± 0.93 L *versus* 4.14 ± 0.78 L for women). This suggests that the CNN model’s training on a diverse dataset may help mitigate biases introduced by external anatomical markers such as chest shape. These findings highlight the importance of selecting appropriate lung volume determination methods, particularly in studies investigating sex-related differences in lung diseases.

The smallest difference in lung volume in our study was found for the comparison between the CNN and CT methods. Thus, a CNN-based approach for the assessment of lung volume from conventional radiographs might be a promising tool for the determination of the mean dark-field coefficient, especially when considering that the approach proposed by Pierce et al [12], which was used in most previous studies on clinical dark-field radiography [3, 4, 9], had only been ascertained in a small cohort of 35 participants of whom CT was only performed in four. Also, the CNN approach will allow for the calculation of the focal dark-field coefficient, which will be helpful for the assessment of lung disease with focal manifestation, not possible with the method by Pierce et al 12]. However, the CNN method also comes with a disadvantage: foreign bodies result in a failure of the CNN, and the lung volume in those patients cannot be obtained with this method. Also, the slope of the regression line for the correlation between CT and CNN as well as PFT and CNN was smaller than 1 (CT 0.54; PFT 0.65), suggesting a bias depending on the participant’s lung volume with an overestimation of lung volume determined by the CNN method in smaller lungs and an underestimation in bigger lungs, when considering PFT and CT as reference standard.

Even though the methods’ differences in lung volume forbid a quantitative comparison of mean dark-field coefficients calculated with different methods, the correlations between lung volumes determined with all four methods are high (*ρ* = 0.78–0.88). All four approaches may be valid as long as the lung volume determination method remains the same within one study. However, in our opinion, CR-based methods are more desirable for lung volume assessment in the context of mean dark-field coefficient calculation, as both dark-field image and conventional image are acquired at the same breathing state and therefore biases due to differences in inhalation depth are eliminated. While the absolute lung volume differs between the different methods, we found a high positive correlation for all methods when compared to each other. This should allow a cross-comparison by scaling of the obtained volumes. Multiplying with an adjustment factor should enable a wider comparability between different methods and could lead to a generalized volume estimate, adjusted for the used method. However, this factor needed to be determined in larger cohorts and is subject to future research.

Finally, it is important to note that our study did not exclude emphysema patients, as doing so would have significantly reduced the available cohort. Emphysema leads to an increase in lung volume, not only in pulmonary function testing (PFT) but also in CT- and CR-based volume assessments. This suggests that hyperinflation affects all structural lung volume determination methods similarly, allowing for a valid comparison across techniques. While emphysema is known to impair lung function, our study focused on structural lung volume rather than functional parameters. Future research could explore whether deep learning-based segmentation methods, such as CNN, are influenced by specific lung pathologies and whether emphysema-related overinflation introduces systematic biases in automated lung volume assessment.

This study has limitations. First of all, CT imaging was performed under routine clinical conditions and not as part of a study, and therefore, as mentioned above, breathing instructions did differ between CT, CR, and PFT. Also, the CNN was trained on synthetic radiographs derived from CT images. This was to ensure that the breathing state was the same for CT and radiographs. However, the fact that CT images (and thus synthetic radiographs) were acquired in supine position might have led to a bias, as the CNN was then applied to radiographs acquired in standing position. Since most previous dark-field studies have relied on CR-based lung volume assessment, switching to CNN-derived volumes could influence study outcomes, particularly in cases such as emphysema staging or sex-based differences in lung function. As our results suggest, CNN-based volume estimation aligns more closely with CT, which may improve the accuracy of mean dark-field coefficient calculations. However, future studies should investigate whether CNN-derived volumes introduce systematic differences in disease classification compared to traditional CR-based approaches. Establishing an adjustment factor or calibration method could help standardize results across different volume determination techniques.

In conclusion, our results showed that lung volume and, therefore, the mean dark-field coefficient calculation, are highly dependent on the method used, especially due to different positioning and inhalation depths.

## Data Availability

The datasets used and/or analyzed during the current study are available from the corresponding author on reasonable request.

## Abbreviations

CR: Conventional radiography
CT: Computed tomography
PFT: Pulmonary function testing
TDF: Total dark-field signal
TLC: Total lung capacity
